# Author Correction: Allelic variation at a single locus distinguishes spring and winter faba beans

**DOI:** 10.1038/s41588-026-02574-2

**Published:** 2026-03-23

**Authors:** Hailin Zhang, Alex Windhorst, Elesandro Bornhofen, Zuzana Tulpova, Petr Novak, Jiri Macas, Hana Simkova, Marcin Nadzieja, Jung Min Kim, Dustin Cram, Yongguo Cao, David J. F. Konkin, Olaf Sass, Gregor Welna, Axel Himmelbach, Martin Mascher, Wolfgang Link, Soon-Jae Kwon, Tae-Jin Yang, Stig Uggerhøj Andersen, Murukarthick Jayakodi

**Affiliations:** 1 Leibniz Institute of Plant Genetics and Crop Plant Research (IPK) Gatersleben, Seeland, Germany; 2 Georg-August-University Göttingen, Department of Crop Sciences, Göttingen, Germany; 3 Center for Quantitative Genetics and Genomics, Aarhus University, Aarhus, Denmark; 4 Institute of Experimental Botany of the Czech Academy of Sciences, Olomouc, Czech Republic; 5 Biology Centre, Czech Academy of Sciences, České Budějovice, Czech Republic; 6 Advanced Radiation Technology Institute, Korea Atomic Energy Research Institute, Jeongeup, Korea; 7 National Research Council Canada, Saskatoon, Saskatchewan Canada; 8 Norddeutsche Pflanzenzucht Hans-Georg Lembke KG, Holtsee, Germany; 9 German Centre for Integrative Biodiversity Research (iDiv) Halle-Jena-Leipzig, Leipzig, Germany; 10 Department of Agriculture, Forestry and Bioresources, Plant Genomics and Breeding Institute, Research Institute of Agriculture and Life Science, College of Agriculture and Life Sciences, Seoul National University, Seoul, Republic of Korea; 11 Department of Molecular Biology and Genetics, Aarhus University, Aarhus, Denmark; 12 Texas A&M AgriLife Research and Extension Center, Texas A&M University System, Dallas, TX USA; 13 Department of Soil & Crop Sciences, Texas A&M University, College Station, TX USA

**Keywords:** Genome assembly algorithms, Genome-wide association studies, Gene expression

Correction to: *Nature Genetics* 10.1038/s41588-026-02524-y, published online 10 March 2026.

In the version of the article initially published, in the Extended Data Fig. 3 legend, “The motif of telomeric region is TTAGGG” should have read “The motif of telomeric region is TTTAGGG”. This correction has been made to the HTML and PDF versions of the article.

